# Unveiling the Diet of Elusive Rainforest Herbivores in Next Generation Sequencing Era? The Tapir as a Case Study

**DOI:** 10.1371/journal.pone.0060799

**Published:** 2013-04-01

**Authors:** Fabrice Hibert, Pierre Taberlet, Jérôme Chave, Caroline Scotti-Saintagne, Daniel Sabatier, Cécile Richard-Hansen

**Affiliations:** 1 Direction Etudes et Recherches, Office National de la Chasse et de la Faune Sauvage, Kourou, French Guiana, France; 2 Laboratoire d'Ecologie Alpine, CNRS, UMR 5553, Université Joseph Fourier, Grenoble, France; 3 Laboratoire Evolution et Diversité Biologique, CNRS, UMR 5174, Université Paul Sabatier, Toulouse, France; 4 INRA, UMR 0745 EcoFoG, Kourou, French Guiana, France; 5 IRD, UMR AMAP, Montpellier, France; Centro de Investigación y de Estudios Avanzados, Mexico

## Abstract

Characterizing the trophic relationships between large herbivores and the outstanding plant diversity in rainforest is a major challenge because of their elusiveness. This is crucial to understand the role of these herbivores in the functioning of the rainforest ecosystems. We tested a non-invasive approach based on the high-throughput sequencing of environmental samples using small plant plastid sequences (the trnL P6 loop) and ribosomal ITS1 primers, referred to as DNA metabarcoding, to investigate the diet of the largest neotropical herbivore, the lowland tapir. Sequencing was performed on plant DNA extracted from tapir faeces collected at the Nouragues station, a protected area of French Guiana. In spite of a limited sampling, our approach reliably provided information about the lowland tapir's diet at this site. Indeed, 95.1% and 74.4% of the plant families and genera identified thanks to the trnL P6 loop, respectively, matched with taxa already known to be consumed by tapirs. With this approach we were able to show that two families and eight new genera are also consumed by the lowland tapir. The taxonomic resolution of this method is limited to the plant family and genera. Complementary barcodes, such as a small portion of ITS1, can be used to efficiently narrow identifications down to the species in some problematic families. We will discuss the remaining limitations of this approach and how useful it is at this stage to unravel the diet of elusive rainforest herbivores and better understand their role as engineers of the ecosystem.

## Introduction

Ongoing environmental threats such as forest fragmentation and unsustainable game hunting, in addition to the pervasive role of global changes, all lead to a decline of the large tropical rainforest fauna [1], [2]. Understanding the role of the large vertebrate herbivores in the functioning of the rainforest ecosystems is an important issue in ecology and conservation because these animals are thought to be important ecosystem engineers [3]–[9]. In spite of significant advances in the study of large vertebrate herbivores (e.g. [7], [10], [11]), the lack of qualitative and quantitative information on the relationships with the other organisms of their environment remains a major impediment to fully identifying their ecological role and to foresee the consequences of their local extinction.

Large rainforest herbivores are elusive animals, and the study of their foraging strategies must rely on invasive analyses or other indirect methods. These methods classically include browsing signs analysis (e.g. [12]), macroscopic and microscopic analysis of gut (e.g. [13], [14], [15]) and of faecal contents (e.g. [16], [17]). These techniques are often limited to few species and individuals and usually yield an incomplete quantification of the diet [17], [18]. Consequently, the range of plants impacted by large rainforest herbivores is still improperly known, as well for instance how herbivores select their food, how this food affects their spatial behaviour and resource partitioning [12], [19].

Recent studies have stated that new molecular techniques may constitute robust and accurate approaches complementing the conventional ways of analysing wildlife diet [20], [21], [22]. By combining the identification of consumed items through DNA barcoding [23], [24] and next-generation sequencing (NGS), several authors have recently been able to determine the diet of animal species, both in carnivores (e.g. [22], [25]–[27]) and in herbivores [21], [24], [28]–[31]. To date, this approach has seldom been tested to characterize herbivory in tropical forest ecosystems [18], [32], [33].

The scientific community is very excited about the potential uses of NGS to understand ecological processes. As with any novel technique, the expectations are high but there is limited evidence of how useful this tool really is. This study aims at contributing to our understanding of the real potential and limitations of DNA metabarcoding to understand diets in large tropical rainforest herbivores.

New target DNA fragments, such as the plastid *trnL* intron and its P6 loop, have been recently identified as useful DNA barcodes in case DNA is highly degraded after the passage through the digestive tract of herbivores [34]. Indeed, the P6 loop marker has universal primers in flowering plants, a short size of the target fragment (typically less than 100 bp) and high inter-specific variation in size and sequence [28]. If some *a priori* knowledge of the herbivore's diet is available, plant group-specific primers may also be used to refine the resolution of the analysis [20], [31], [33], [35]. Moreover, the recent development of high throughput sequencing technology [20] now allows the production of thousands of short-length sequences (e.g. [21], [25]). Combining short-length DNA barcodes coupled with next-generation sequencing of environmental samples has been termed "DNA metabarcoding" [36], [37].

We applied the method on the largest neotropical herbivore, the lowland tapir (*Tapirus terrestris* Linnaeus, 1758). Quite recently, our knowledge on the tapir’s diet has been significantly extended for both the fruit and foliar or "browse" diet [17], [18]. This provides a solid knowledge basis for comparison with metabarcoding. Field work was performed at the Nouragues Ecological Research Station, in French Guiana, where a local reference database of plant species DNA barcodes has been developed [38]. Hibert et al. [18] showed that it is feasible to use a classical DNA barcoding (using the plastid DNA trnH-psbA region, of 500 bp on average) of faecal material to identify items of the tapirs’ diet. However, the test was conducted on a limited number of individual plant fibre samples and the molecular approach was far from providing as much information on the diet as the combination of the other classical approaches used.

In the present contribution, we report results based on DNA metabarcoding of tapir faeces, in the hope to yield a better coverage of the diversity of the tapirs’ diet by targeting many more DNA sequences extracted from mixed material (and not only preselected individual plant fibre samples). The tapirs’ diet is known to seasonally vary in relation to fruit availability [14], [17]. We tested whether DNA metabarcoding yields a diet variation between seasons. We finally discuss the consistency of the plant taxa identified through metabarcoding with those indicated by alternative approaches and the remaining limitations of this approach to the understanding of the diet of tropical rainforest herbivores.

## Materials and Methods

### Study site

The tapir faeces were collected in the Nouragues Ecological Research Station (4°05'N, 52°40'W), in French Guiana. This station is included in a 1000 km^2^ protected area, the Nouragues Natural Reserve, 25–430 m above sea level, and is characteristic of the primary rainforest of Northeastern South America ([39], www.nouragues.cnrs.fr). The local flora includes over 1700 angiosperm species, a large proportion of which are endemic of the Guiana shield [40], [41]. Annual rainfall averages 2880 mm. A distinct dry season occurs from September to November (<100 mm per month), followed by a shorter drier period around March. Tree fruiting peaks in March-May and is minimal in August-September [42], [43].

### Faecal samples collection and preparation

Since tapirs are known to preferentially defecate in water in French Guiana (Hibert pers. obs. in 4 captive tapirs, local interviews), we focused our research along the reserve streams in order to maximize the chance to encounter fresh tapir dung piles. The encounter rate was not regular over time and in some months we collected more samples than others. In total we analysed samples from 39 tapir dung piles. For collection methods and stocking conditions, the reader is referred to the details provided in Hibert *et al.* [18]. All necessary permits were obtained for the described field studies. The research program and sampling collection were approved and validated by the Conseil Scientifique Régional du Patrimoine Naturel (CSRPN) and as well by the Conseil Scientifique of CNRS for the studies in the Research area of the Reserve. On average, dry dung piles weighed 178.8 g. (quantiles: 0%: 15.4 g, 25%: 62.8 g, 50%: 180.0 g, 75%: 276.0 g, 100%: 353.9 g). To test the influence of collection and preliminary sorting , we prepared two samples of about 60 mg per dung pile (N = 39; 78 samples). The first sample, hereafter named the "sorted" sample, included only large homogeneous fragments (mainly parts of leaf limbs), while the second, hereafter named the "unsorted" sample excluded them (random smaller fragments). These samples were then separately dry ground with a Mixer Mill MM 200 (Retsh ©, Haan, Germany, frequency: 22 s-1 for 1 min) to prepare a homogenized product mix for further DNA extraction.

### Molecular analyses

#### DNA extraction from faeces

Total DNA was extracted from each of the 60 mg faecal samples with the DNeasy Blood and Tissue Kit (QIAgen GmbH, Hilden, Germany), following the manufacturer’s instructions. The DNA extracts were recovered in a total volume of 250 µL. Mock extractions without samples were systematically performed to monitor possible contaminations.

#### DNA amplification

Quality of the DNA extractions has been checked by electrophoresis on agarose gel (1.4%) before performing DNA amplification on a 100 x diluted DNA extract (4 µL in a final volume of 50 µL) to avoid high rates of PCR inhibitors. The amplification mixture contained 1 U of AmpliTaq® Gold DNA Polymerase (Applied Biosystems, Foster City, CA), 10 mM Tris-HCl, 50 mM KCl, 2 mM of MgCl2, 0.2 mM of each dNTP, 0.1 µM of each primer, and 0.005 mg of bovine serum albumin (BSA, Roche Diagnostic, Basel, Switzerland). The mixture was denatured at 95°C for 10 min, followed by 45 cycles of 30 s at 95°C, and 30 s at 55°C; as the target sequences are usually shorter than 100 bp, the elongation step was removed. Samples were amplified using two primer-pairs (Table 1). The first pair (*g* and *h*) amplifies the P6 loop region of the *trnL* (UAA) intron across flowering plants [34].

**Table 1:** Primers used in this study.

Name	Primer sequence (5'–3')	Reference
g	GGGCAATCCTGAGCCAA	Taberlet *et al*. 2007
h	CCATTGAGTCTCTGCACCTATC	Taberlet *et al*. 2007
ITS1-F	GATATCCGTTGCCGAGAGTC	Baamrane *et al.* 2012
ITS1Sap-R	CGACGCGAATCGCGTCAAGGA	this study

In order to increase the resolution of the analysis, and test the feasibility of a two-tiered approach using metabarcoding techniques, we also used another primer pair. This new primer-pair targets the first internal transcribed spacer (ITS1) of nuclear ribosomal DNA, for the Sapotaceae (ITS1-F and ITS1Sap-R). This plant family was chosen, since it is abundant in Amazonia, and its fruits are known to be consumed by tapirs [18]. All primers were modified by the addition of specific tags on the 5′ end to allow the assignment of sequence reads to the relevant sample [24]. All the PCR products were tagged identically on both ends. These tags were composed of CC on the 5' end followed by nine variable nucleotides that were specific to each sample. The nine variable nucleotides were designed using the oligoTag program (www.prabi.grenoble.fr/trac/OBITools) with at least three differences among the tags, without homopolymers longer than two, and avoiding a C on the 5' end. All the PCR products from the different samples were first titrated using capillary electrophoresis (QIAxel, QIAgen GmbH, Hilden, Germany) and then mixed together, in equimolar concentrations, before the sequencing.

#### DNA sequencing

The sequencing was carried out on an Illumina/Solexa Genome Analyzer IIx (Illumina, San Diego, California), using the Paired-End Cluster Generation Kit V4 and the Sequencing Kit V4 (Illumina, San Diego, California), following manufacturer's instructions. A total of 108 nucleotides were sequenced on each extremity of the DNA fragments.

#### Sequence analysis and taxon assignation

The sequence reads were analyzed using the OBITools suite of bioinformatics routines (www.prabi.grenoble.fr/trac/OBITools). First, the forward and reverse reads corresponding to a single molecule were aligned and merged using the solexaPairEnd program, taking into account quality data during the alignment and the consensus computation. Then, primers and tags were identified using the ngsfilter program. Only sequences with perfect match on tags and a maximum of two errors on primers were taken into account. The amplified regions, excluding primers and tags, were kept for further analysis. Strictly identical sequences were clustered using the obiuniq program, keeping the information about their distribution among samples. Sequences shorter than 10 bp for the P6 loop and 40 bp for the ITS region specific of the Sapotaceae, or containing nucleotides other than A, C, G and T, or with occurrence lower or equal to 10 were excluded using the obigrep program. Taxonomic assignation of the clusters was achieved using the ecoTag program [28].

EcoTag relies on an exact global alignment algorithm [44] to find highly similar sequences in the reference databases. These databases were built by extracting the P6 loop of the *trnL* intron or the ITS1 fragment from the European Molecular Biology Laboratory (EMBL) nucleotide library (release 107) using the ecoPCR program [45]. The P6 loop and the ITS1 databases contain 11,905 and 2596 unique sequences extracted from 54,239 and 5650 EMBL entries, respectively. EcoTag assigned a unique taxon to each unique sequence amplified from tapirs’ faeces. This unique taxon corresponds to the last common ancestor node in the National Center for Biotechnology Information (NCBI) taxonomic tree of all the taxids annotating the sequences of the reference database that best matched on the whole length of the query sequence.

#### Dataset trimming

**Figure 1:**
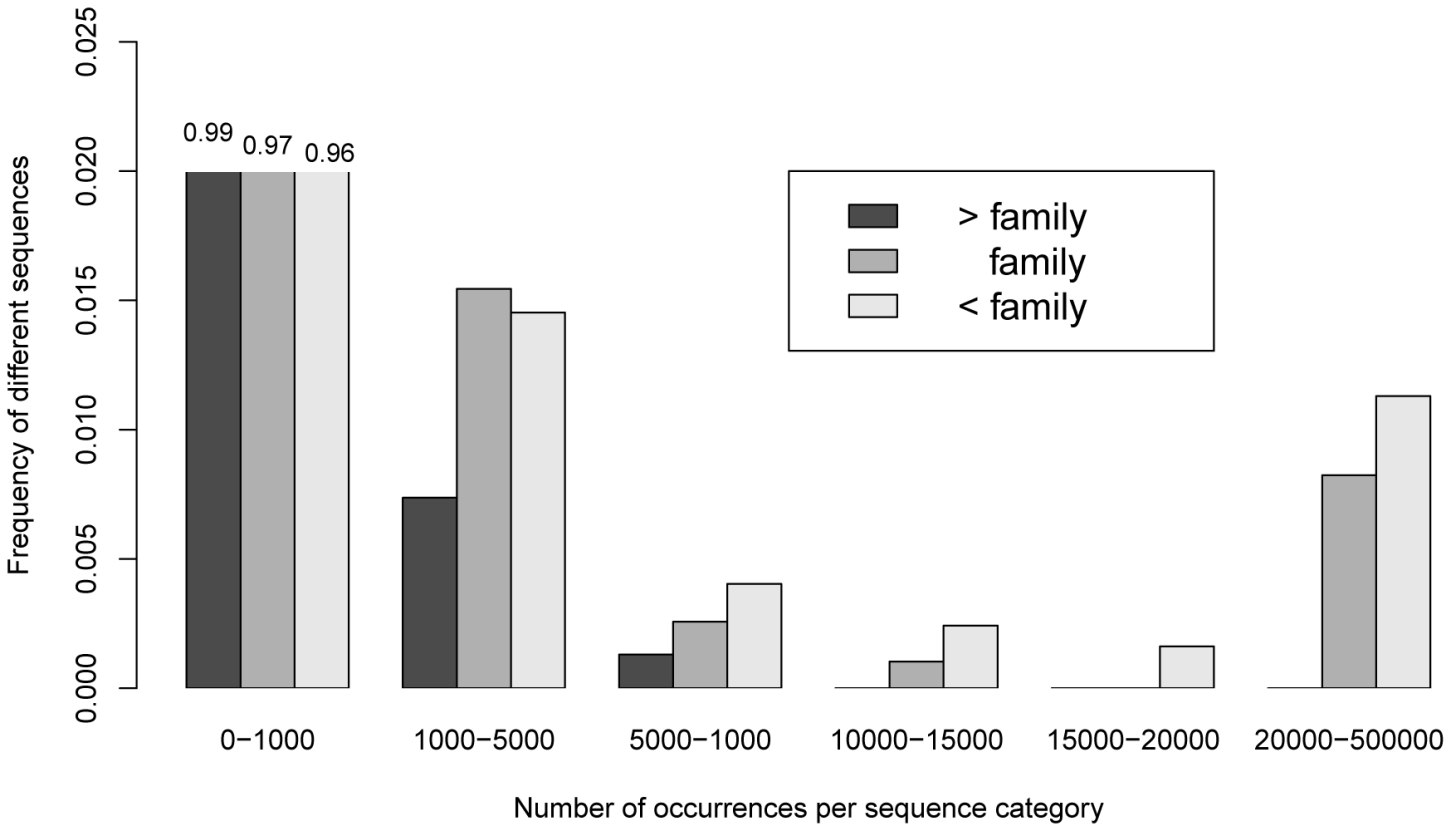
Abundance distribution of the P6 loop sequences at different taxonomic levels. "< family": sequences identified at a finer taxonomic resolution than the family, "family": sequences identified at the family level only, "> family": sequences that could not be identified up to the family level. N = 5488 unique sequences longer than 10 bp and with occurrence higher or equal to 11.

The sequencing produced 70552 unique P6 loop sequences longer than 10 bp and 15162 unique ITS sequences longer than 40 bp (Table 2). A number of these sequences could not be assigned to any known taxon. The P6 loop sequences that could not be identified up to the family level (41.9%) were less frequent than those identified at a finer taxonomic resolution (Figure 1) and probably resulted from PCR artefacts.

**Table 2:** Number of sequences assembled by NGS of vegetal DNA extracted from tapir faeces (N = 39, 79×60 mg of faecal product), using plant universal primers targeting the P6 loop region of the trnL (UAA) intron and primers specific for the Sapotaceae family, targeting the first internal transcribed spacer (ITS1) of nuclear ribosomal DNA.

Primer pair	g / h ^(1)^	ITS1-F / ITS1Sap-R ^(2)^
Number of properly assembled sequences	3246036	77884
Number of unique sequences	70588	15183
Number of unique sequences longer than 10 ^(1)^ or 40 ^(2)^ bp	70552	15162
Number of unique sequences longer than 10 ^(1)^ or 40 ^(2)^ bp, and with occurrence higher or equal to 11	5488	257
Number of unique sequences after cleaning of the artefacts	117	12
Corresponding total number of sequences analysed	2598249	15614

To eliminate the artefact sequences, we automatically removed sequences that did not correspond to any plant sequences present in the EMBL database (homology < 0.95) except the sequences that had a perfect match with at least one of the DNA barcodes of the Nouragues databases that contain 158 species for the trnL intron and 75 species for the ITS1 region [38]. We also removed every sequence for which there was a very similar sequence (only one base different) but always in higher occurrence, since it was assumed to be an erroneous copy of the latter. Finally, we only kept for further analysis the P6 loop and ITS sequences with a total number of occurrences higher or equal to 480 or 59, respectively. This corresponds to 1/1000 or 1/100 of the most common sequences for the P6 loop and for the ITS1, respectively.

### Statistical analyses

#### Importance of preconditioning the samples

To test whether the pairs of samples from same dung piles had more similar compositions than random samples from any dung piles, we first calculated the proportional sequence turnover βP [46] between pairs of samples using the presence-absence table of the P6 loop sequences in the dung pile samples. βP is calculated as the total sequence diversity (gamma) minus the mean sequence diversity (alpha) divided by gamma [46]. Then, we used a Monte-Carlo procedure with 1000 simulations to compare the mean genetic distance between samples from same dung piles to the distribution of mean genetic distances calculated between a same number of samples from random dung piles. We also tested whether the sorted samples (fewer but larger homogeneous fragments) yielded less diverse sequences than the unsorted samples.

#### Diet richness and sampling effort assessment

We used accumulation models to assess the completeness of the inventory method relative to the sampling effort invested, in terms of number of dung piles sampled, and to estimate the richness of the tapirs’ diet in the study area. The analysis was performed with the unsorted samples because they were more diverse (see Results). The richness of the diet was estimated in terms of number of both P6 loop sequences and identified families. We generated rarefaction curves using 500 Mao Tau randomizations [47] with EstimateS Version 8.2 [48]. Total richness was estimated by the Chao2 estimator [49].

#### Seasonal variation in the diet composition

We measured the relative dependency of the diet composition patterns with season by running a Between-Class Analyses (BCA) [50] (1) on the presence-absence table of all the P6 loop sequences and (2) on the presence-absence table of the sequences of the identified genus in the dung piles using the months of sample collection as the grouping classes. Both BCAs yielded a between-months inertia percentage. We further tested these ratios with 9999 Monte-Carlo randomizations.

Telemetry studies [51] and field observations of presence indices (Hibert pers. obs) suggest that tapirs may spend a few days in some part of their home-range before moving to exploit another part. In the study area, the vegetation structure and nature varies spatially at the landscape scale [52]. If tapirs do not travel much throughout their whole homerange within the digestion time, the composition of their dung may reflect to some extent the spatial variation in the availability of consumed plants. We used a Mantel test based on Pearson's product-moment correlation between the composition distance matrix and the matrix of Euclidian spatial distance between the sampled dung piles. Statistical significance was assessed by a Monte-Carlo permutation test with 9999 simulations. The composition distance matrix between samples was computed with the βP measure applied on the presence-absence table of the P6 loop sequences in the dung piles.

The seasonal variations of the occurrences of the P6 loop sequences identified at the genus level were then analysed in regard to the mean monthly abundance of macroscopic fruit residuals of the same genus found in the same dung piles [18]. The coincidence of the sequence abundance peaks in the faeces with the fructification period was tested with a Pearson's correlation test after a log+1 transformation of the data.

The statistical analyses were performed with the R software, using the *ade4* package [53]. The Mantel test was calculated with the function *mantel.rtest* and the Between-Class Analysis with the function *between*.

## Results

### Overall diversity of plant items retrieved in the faeces

**Figure 2:**
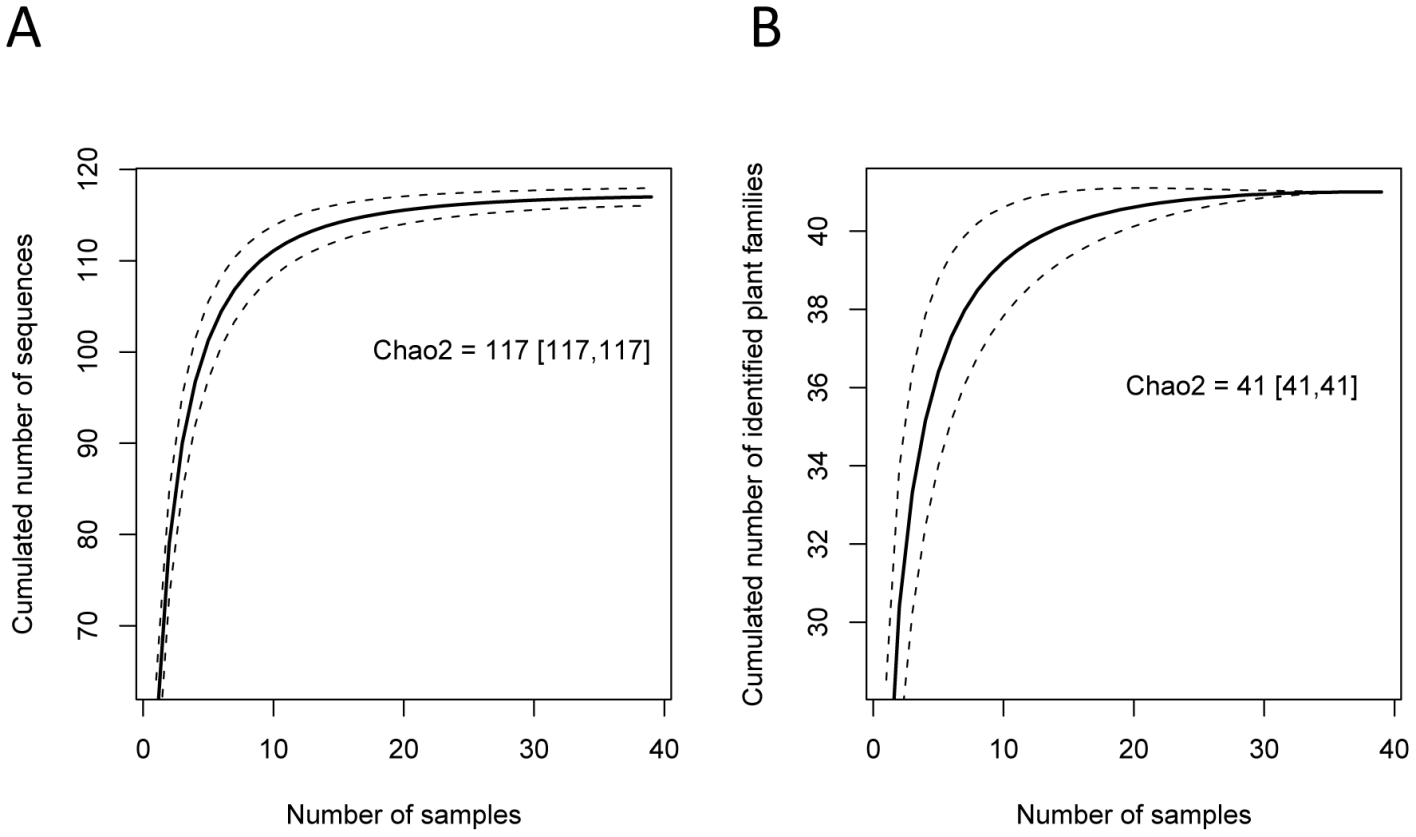
Accumulation curves of plant richness in the sampled tapir dung piles using the P6 loop marker. a) Cumulated richness of the plant DNA sequences (P6 loop), b) Cumulated richness of the identified plant families.

The NGS run produced 70588 and 15183 unique sequences with the *g/h* and ITS1 primers respectively. After cleaning the supposed artefacts, we ended with 117 P6 loop sequences, with a total count of 2598249 sequences corresponding to 80% of the initial number of reads, and 12 ITS sequences (20% of the initial number of reads) from the 39 dung samples (Table 2). The cumulated number of P6 loop sequences and corresponding identified taxa both reached an asymptote suggesting that continued sampling of tapir faeces would not bring much more information about the richness of the diet (Figure 2).

### Importance of the faecal sample treatment

DNA extraction, amplification and sequencing succeeded in every sample, irrespective of the treatment. Samples from same dung piles had more similar compositions than random pairs of samples, with a mean composition distance between samples from same dung piles was, indeed, significantly smaller than those simulated between random samples (Monte-Carlo permutation test, p  =  0.003). This result is confirmed by a positive correlation between the richness of the P6 loop sequences from the unsorted and sorted samples (Pearson's product-moment correlation, r  =  0.54, p  =  0.001). As expected, the sequence richness was on average significantly higher in the unsorted samples (58.1 ± 14.2 (sd)) than in the sorted ones (53.1 ± 14.6 (sd)) (difference  =  5.1, 95%CI  =  [0.2,10.0]) for a same amount of treated faecal material.

### Main identified taxa

**Figure 3:**
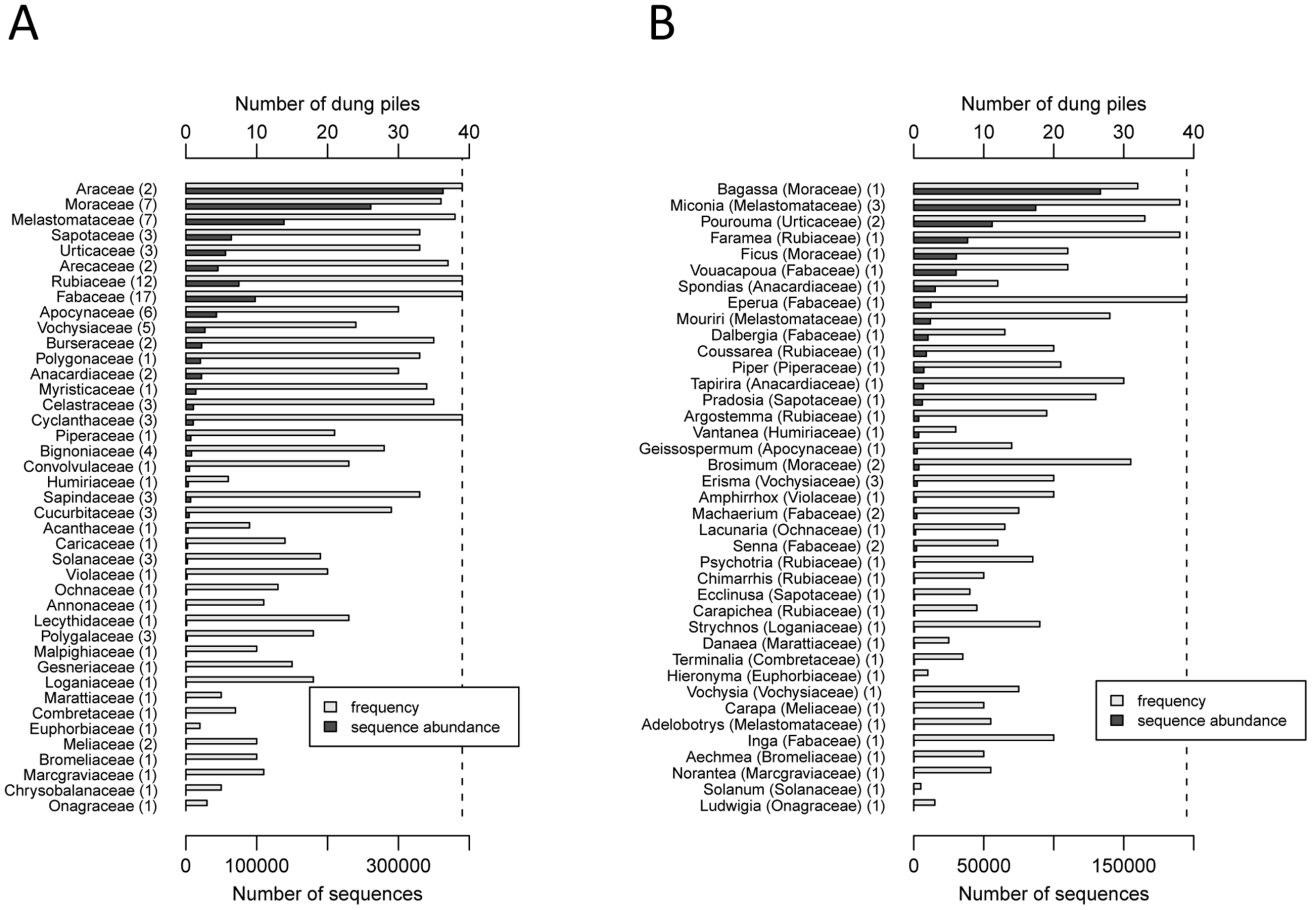
Total abundances and frequencies of the P6 loop sequences of the most abundant plant taxa identified in the sampled tapir dung piles. a) Identified families; b) Identified genera. Figures into brackets indicate the number of different sequences per taxon.

The barcode reference databases allowed to identify 113 (96.6%) and 47 (40.2%) of the 117 unique P6 loop sequences up to the family and genus levels, respectively. They were assigned to 41 families, 39 genera and 3 species (Table S1, Figure 3). In each dung pile sample, we identified on average 24 families (sd  =  5.8, min  =  11, max  =  34) and 10 genera (sd  =  2.8, min  =  4, max =  16). Further identification of 10 more species could be achieved in the case of some genera with only one species listed in the Reserve (Table S1). The most repeated sequence matched perfectly with three different genera of the Araceae family. The most frequent P6 loop sequences also matched well the most frequent genera known to be found in the dung piles (Pearson's product-moment correlation between the number of repetitions and frequency of the sequences, r  =  0.62, p < 0.001, Figure 3).

As expected, the ITS1 primers specially designed to amplify the Sapotaceae family yielded a refined determination resolution, with 9 (75.0%) and 8 (66.7%) of the 12 unique sequences directly attributed to a genus and to a species, respectively, after confrontation to the EMBL database. Coupling with the local ITS database of the Nouragues allowed to further refine the identification of one unidentified sequence to one more species. The list of identified taxa is given in Table 3.

**Table 3:** List of plant taxa identified in 39 tapir dung of the Nouragues Reserve using the ITS markers.

Family	Genus	Species
Burseraceae	Protium	P. subserratum (Engler) Engler^N^
Sapotaceae	Chrysophyllum	C. eximium Ducke
		C. sanguinolentum (Pierre) Baehni^N^
	Micropholis	M. cayennensis Pennington^N^
	Pouteria	P. egregia Sandwith *
		P. fimbriata Baehni*^N^
		P. gongrijpii Eyma^N^
		P. guianensis J.B. Aublet
	Pradosia	P. ptychandra (Eyma) Pennington
	Sarcaulus	S. brasiliensis (A.L. De Candolle) Eymas^N^

Presence of the taxa in the region was checked in Funk *et al*. (2007). Species names followed by a "*" and by a "^N^" are for sequences further identified at the species level thanks to the local database for the Nouragues Reserve (Gonzalez *et al.* 2009) and not yet listed in the diet of the lowland tapir throughout its range (comparison with review by Hibert *et al.* 2011), respectively.

### Seasonal variation of diet composition

**Figure 4:**
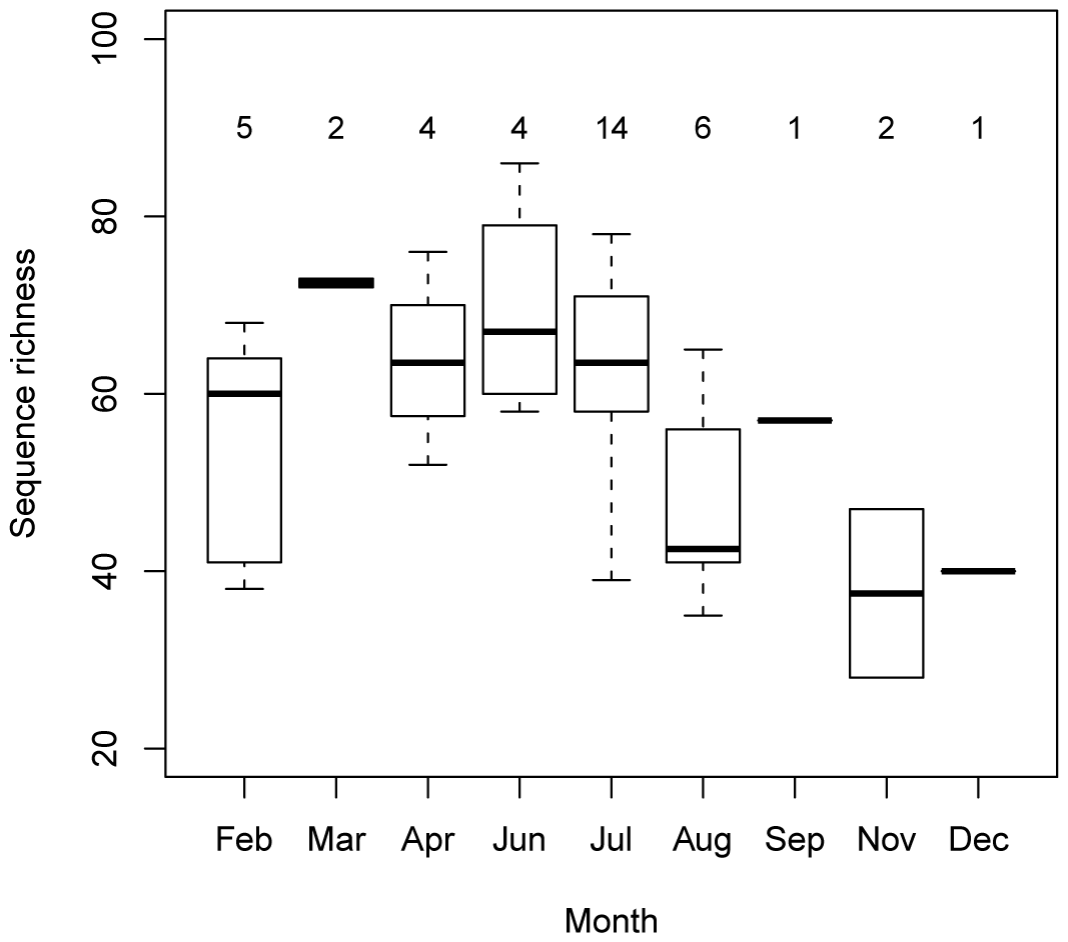
Box plot of the richness per month of the *trnL* P6 loop plant sequences (length > 10 bp) detected in the tapir dung piles, using unsorted samples. The figures on top indicate the number of samples per month.

**Figure 5:**
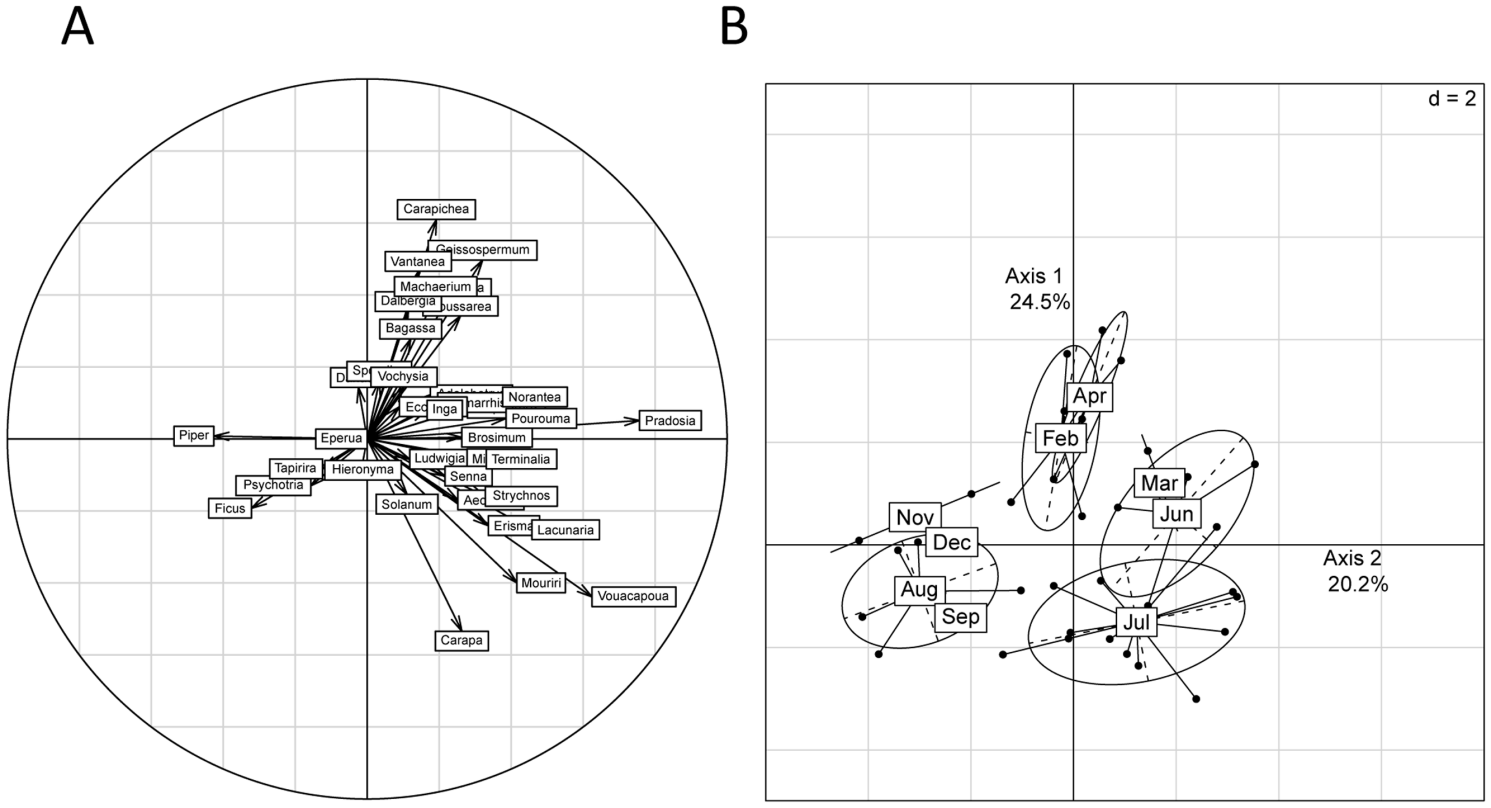
Results of the between-class analysis of the presence-absence table of the identified plant genera in the tapir dung piles between the months of sample collection. a) Correlation circle of the plant genera in the two first axes of the BCA; b) Distribution of the dung samples in the first plan of the BCA. The mesh of the grid is 2. The ellipses circumscribe the dung samples for each month. They are centred on the barycentres and the length of the ellipse axes equal to 1.5 time the standard deviation of the coordinates of the projections of the points (samples) on the BCA axes.

We did not detect any relationship between the variation the composition and the spatial distance between the dung piles (Mantel test, r  =  0.09, p  =  0.167). However, the richness of sequences in dung piles peaked between March and July (Figure 4), eventhough no significant difference of sequence richness could be detected between months (Tukey multiple comparisons of means, p > 0.062). The BCAs indicated that 28.9% (p  =  0.001) and 31.1% (p  =  0.001) of the total variation in the diet composition measured by present P6 loop sequences and identified plant genera in the dung piles, respectively, was affected to the between-months variation, suggesting a seasonal shift in the diet. Both BCAs primarily (first axis) opposed samples collected in March, June and July, containing rather sequences attributed to *Pradosia*, *Vouacapa, Lacunaria*, *Mouriri*, *Strychnos*, *Pourouma* and *Norantea* to those collected from August to December rather with *Ficus* and *Piper* and, secondarily (second axis), distinguished samples mainly collected between February and April with *Carapichea*, *Geissospermum*, *Machaerium*, *Vantanea*, *Argostemma*, *Dalbergia*, *Coussarea* from those collected between July and September with *Carapa*, *Vouacpa*, *Mouriri* and *Ficus* (Figure 5).

The sequence occurrence of the identified genera in the dung piles varied much along the year, most of them presenting seasonal peaks, whereas some others were found all year round (*Faramea*, *Miconia*, *Aechmea*, *Norantea*). In three genera (*Geissospermum*, *Lacunaria*, *Spondias*), the peaking in the P6 loop sequence frequency coincided with the presence of fruit residuals observed in the dung piles (Table 4). Among the 17 genera macroscopically identified by their diaspore residuals in the 39 dung samples, ten were identified using the P6 loop sequence barcode, one (*Moutabea*) was not retrieved at all and six (*Byrsonima*, *Heisteria*, *Micropholis*, *Pouteria*, *Salacia*, *Swartzia*) were observed in too low frequency (repetitions of P6 loop ≤33, ≤361, ≤163, ≤11, ≤163, ≤49 respectively) to be considered as significant. Nevertheless, the use of the ITS marker for the Sapotaceae confirmed the presence (> 1000 repetitions) of both *Micropholis* and *Pouteria* at the same time a case for which the macroscopic fruit residuals were also observed.

**Table 4:** Correlation between the sequence (P6 loop *trnL*) occurrence and the mean monthly abundance of macroscopic fruit residuals of the same genus found in the same dung piles.

Genus	r	p.value	t	df
Carapa	−0.20	0.631	−0.506	6
Ficus	0.40	0.327	1.067	6
**Geissospermum**	**0.82**	**0.013**	3.473	6
**Lacunaria**	**0.82**	**0.012**	3.541	6
Mouriri	0.43	0.284	1.176	6
Pourouma	−0.13	0.756	−0.325	6
Pradosia	0.01	0.983	0.022	6
Psychotria	0.19	0.661	0.461	6
**Spondias**	**0.96**	**0.000**	8.331	6
Tapirira	−0.04	0.918	−0.107	6

Genera with positive significant correlation are indicated in bold.

## Discussion

### Diversity of the diet indicated by the P6 loop marker

The high throughput sequencing technology allowed to produce thousands DNA sequences from every tapir dung pile tested despite the degraded state of faecal material. From these sequences, we discarded a large proportion suspected to be erroneous and the approach seems to yield a lot of waste. Nevertheless, this is not surprising since the production of a very large number of unique sequences with a very low frequency is typical from this type of experiment [22], [27] and results either from degraded template DNA in the faeces, from nucleotide misincorporation during DNA amplification, or from the sequencing process itself [24]. We actually kept not less than 80% of the total properly assembled P6loop sequences and our very conservative way to clean the statistical artefacts during the sequence analysis minimized the risk of considering erroneous sequences. Treating only about 2.34 g of faecal material (39 samples × 60 mg) of faecal material was sufficient to distinguish 117 different plant DNA sequences (P6 loop of the trnL intron) and to circumscribe most of the annual plant sequence richness of tapirs' diet in the study area that was measurable by this approach. Interestingly, the samples, of about only 0.03% of the whole dung pile dry mass, were also sufficiently representative of the whole dung pile composition to indicate the variability among the dung piles (random samples from same dung piles were much more similar in composition than samples from random dung piles). Preliminary sorting of larger homogeneous fragments from faeces did not bring any added value in terms of DNA extraction or sequencing success but unsorted samples yielded a greater richness as expected. We stress that it is not only useless but also counterproductive to preliminary sort the fibres from faeces to isolate the apparently less degraded ones for conducting herbivore diet analysis from faeces content sequencing.

### Taxonomic resolution

Compared to Hibert *et al.* [18], the high throughput sequencing of the P6 loop sequences from the tapir faeces allowed the identification of 51.3% (19/37) of the families and 24.1% (13/54) of the genera identified in the tapirs’ diet. Hibert *et al.* executed this through both classical methods such as the macroscopic analysis of fruit residual in some of the same dung piles analysed here and botanical identification of browsing signs observed in the same area of the Nouragues Reserve during the same period. Nevertheless, 55.6% (10/18) of the families not retrieved here had only been found by Hibert *et al.* [18] in French Guiana and might not be plants very frequently eaten by tapirs. Some plants, consumed as fruits, may have been undetected with the *trnL* P6 loop barcode because it targeted plastid DNA that is usually rarer in fruit organs [28].

Only 40.2% of the P6 loop sequences were identified to the genus level, which is much less well resolved than in the study by Soininen *et al.* [29] for the diet of Norwegian rodents for a comparison (they obtained > 75%). We checked that 87.4% and 69.2% of the 159 families and 643 genera, respectively, of the flowering photosynthetic plants listed in the Nouragues Reserve (http://www.nouragues.cnrs.fr/plant.html, accessed March 2011) had at least one species with the *trnL* (UAA) intron P6 loop sequenced in the EMBL database. Among the 20 non-referenced families, only four (Blechnaceae, Dichapetalaceae, Erythroxylaceae and Thelypteridaceae) have already been listed in the diet of tapirs [18]. Most of the remaining families are small plants, that could hardly be a resource for the tapir, such as small, often epiphytic or epilithic, herbaceous ferns (Grammitidaceae, Hymenophyllaceae, Oleandraceae, Nephrolepidaceae, Schizaeaceae, Vittariaceae) and rare plants or localized trees or lianas (Agavaceae, Hernandiaceae, Thymeleaceae, Trigoniaceae, Ixonanthaceae, Rhabdodendraceae). The local DNA barcoding database [38] allowed us to refine the determination of 14 sequences to the genus level.

The incompleteness of the reference databases, however, explained only part of the low taxonomic resolution since among the 19 plant families with undetermined genus, 15 included genera from the Reserve with the *trnL* (UAA) intron sequenced in the EMBL. The low taxonomic resolution for 11 of these families, and for at least 6 more families (among 41), was explained by shared sequences between several genus. As an example, five genera of the Sapotaceae family carried the same P6 loop sequence.

As a result, tapirs may consume plant species with a low or no variation of the P6 loop. In such plant groups, in temperate regions [24], only closely related species are usually not resolved [34]. In our study, however, only three P6 loop sequence could be identified below the genus level by comparison with the EMBL database. We could check that both *Spondias* and *Hieronyma* had only one species listed in the Nouragues reserve. *Ludwigia* has several species in the reserve but two different species are sequenced in the EMBL and they differ, so the molecular marker appears to have a sufficient resolution to distinguish between the species in this genus. We believe that the species identification in these three cases can then be validated.

The low taxonomic resolution seems not limited to our study since when testing their database developed for the tropical trees of the Nouragues Reserve, Gonzalez *et al*. [38] could unambiguously identify only 63% of the genera using the full *trnL* intron. This resolution power is much lower than expected for a comprehensive database in temperate regions (90% according to Valentini *et al*. [24]). Consequently, it is likely, that the shorter P6 loop of the *trnL* intron is even less efficient in a tropical context to discriminate plant genera. The shared sequences between genera may be explained by a higher rate of lineage diversification and frequent explosive radiations in the tropics compare to temperate ecosystem [38].

Our study stresses the interest of combining complementary markers (see the two-tiered approach of Newmaster *et al.* [54] such as the internal transcribed spacer of the nuclear ribosomal genes to identify the consumed species (e.g. [33]). Indeed, in our study 9 different Sapotaceae species could be attributed to 75% of the sequences targeted by the ITS marker. Although the primers were designed for Sapotaceae, they are not fully specific for this family and also allowed the amplification of one species in the Burseraceae family. Therefore, further development of markers such as in ITS appears promising for gaining precision on the consumed taxa.

### Main plant taxa identified

We can not totally discard the possibility of detecting traces of external DNA contaminating the dung pile sample after defecation with a method as sensitive as NGS. There are different contamination ways such as faecal cross-contamination mediated by dung arthropods that are visiting different piles or contamination by pollen grains from plant species with biparental cytoplasmic inheritance (see [55]) carrying non-diet chloroplast DNA. Nevertheless, we feel that the risk of faecal cross-contamination mediated by phytophageous and coprophageous organisms may be higher in the case of dung deposited on *terra firme* where dung beetles and earth worms and other litter arthropods are very active and the water may also have limited the penetration from anemophylous pollen. Another possible artefact would be the detection of DNA from pollen desposited on the leaves browsed by the tapirs [56]. Nevertheless, the quantity of pollen that might have been deposited on the dung pile is very low in comparison to the faecal material and so is the amount of possible pollen DNA compared to the amount of plant DNA treated. We chose a highly conservative way to select the useful sequences and this included to not consider the less abundant sequences. If there were any pollen DNA sequences produced, we believe that these would certainly be among the discarded sequences.

Our metabarcoding approach identified two plant families to date not known to be consumed by the lowland tapir: the Marcgraviaceae, with the genus *Norantea*, and the Marattiaceae, with the genus *Danaea*, and seven additional new genera: *Terminalia* (Combretaceae), *Hieronyma* (Euphorbiaceae), *Adelobotrys* (Melastomataceae), *Aechmea* (Bromeliaceae), *Dalbergia* (Fabaceae), *Vantanea* (Humiriaceae), *Chimarrhis* and *Argostemma* (Rubiaceae). *Argostemma*, however, is a genus naturally absent from the Guianas and this misidentification may result from the absence of the actually consumed Rubiaceae genus in the EMBL database that carries the same P6 loop. The same risk of misidentifation is possible for all the plant taxa that have not been sequenced in the EMBL yet and this is a major limitation of the approach using only this incomplete reference database.

However and despite a relative low taxonomic resolution in our study case, the metabarcoding approach using the P6 loop approach was well validated by the high concordance with the literature on lowland tapir diet. Out of the families and genera identified here, 95.1% (39/41) and 74.4% (29/39), respectively, were already known to be consumed by tapirs [18]. Moreover, our study allowed to retrieve not less than 41.9% and 10.2% of the plant families and genera, respectively, known to be consumed by lowland tapirs throughout their wide range [18]. This efficiency increased when focusing on the plants consumed in French Guiana, since we identified 51.8% (28/54) of the families and 21.9% (25/114) of the genera known to be consumed in this region. In addition, our approach confirmed that tapirs in French Guiana eat plants from eight families known up to now to be consumed only elsewhere.

If we compare with local information brought by botanical analysis of browsing signs and of fruit residuals in dung samples (including some of the samples treated in the present study) [18], our approach identified 18 other plant families and 20 other genera. This confirmed the interest of the metabarcoding to complement classical approaches of elusive mammal diet.

The most frequent and abundant P6 loop sequences identified in the dung piles belong to taxa among the most often cited in the literature [18]. The top five most abundant families were the Araceae, the Moraceae (notably with genera *Bagassa* an*d Ficus*), the Melastomataceae (with *Miconia* and *Mouriri*), the Sapotaceae (with *Pradosia*) and the Urticaceae (with *Pourouma*). These families, together with the Fabaceae (with *Vouacapoua* and *Eperua*), the Rubiaceae (with *Faramaea*), the Cyclanthaceae, and the Arecaceae were also found in most, if not every, dung pile, all year round. The ITS barcoding also confirmed the appetence of tapirs for Sapotaceae, known to be consumed both as fruits and browse [14], [17], [18]. They consumed at least five of the eight genera, and nine of the 64 species listed in the Nouragues Reserve. We could retrieve four of the 12 Sapotaceae species known to be eaten by the lowland tapir throughout its range and extended this list to five more species.

### Signature of a seasonality in the diet

Several authors have stressed that the frequency of the occurrence of the prey DNA sequences in the faeces gives a biased indication of the quantity of ingested material [20], [24], [25], but may broadly reflect the relative contributions of the used items both in carnivore and herbivore species [25], [26], [30]. Our study of a free ranging wild and elusive animal, in the natural conditions of the rainforest, is far from meeting the minimum controlled conditions to validate the quantitative information on the diet brought by the metabarcoding of faecal material and it has not this pretention. However, we could check here that abundant sequences correspond to plant taxa among the best known to be used by tapirs as food (cf. above). Rayé *et al.* [21] used a two season comparison of the frequencies of P6 loop sequences in the faeces to conclude to a seasonal evolution of the diet of chamois. Our dung samples collected all year round also displayed clear seasonal variations in both the presence and the abundance of some plant sequences, while some others seemed to be consumed all year round, such as *Faramea*, *Miconia*, *Aechmea*, *Norantea*, and might constitute regular browse forage.

The seasonality of the tapirs’ diet also clearly appeared when considering the monthly DNA sequence richness. The observed pattern is very similar to the variation of the relative number of fruiting species around the year [42], despite an important offset towards the end of the fruiting season. As most of the species have short delayed germination, this offset could be explained by a higher amount of young seedlings browsed by tapir. Interestingly, in the cases of *Geissospermum*, *Lacunaria* and *Spondias*, known to be eaten as fruits by the tapirs [18], the abundance of sequence peaked concomitantly with the peaking fruit season. This suggests that the P6 loop can, in a few cases, provide a good signature of some fruits consumed by tapirs, but it can also miss many of them. This is not surprising since we targeted chloroplast DNA which is usually much less abundant in fruits than in leaves and stems. The absence of significant correlation between the abundances of DNA sequences and observed fruit residuals in the faeces in some taxa may also be due to the fact that their seeds had been too much degraded by mastication and digestion or spat out by tapirs for the fruits to be macroscopically identified. If the P6 loop is certainly far to be the best marker to identify the plants eaten as fruits, it is, however, very interesting to point at possible browsed candidate taxa, the browsed part being the less investigated in tapirs and rainforest herbivores in general.

## Conclusions

The DNA of plants found in the faeces is known to be highly degraded by digestion and we opted for using the short P6 loop of the plastid *trnL* intron associated to high throughput sequencing in order to maximise the chance to capture short plant DNA fragments in the faeces. This was successful since we could retrieve DNA from more than 50 different plants in every faecal sample. However, a major limitation to this approach is that the taxonomic resolution is mainly limited to the family or the genus, both because several species can have the same sequence and because the reference database are not yet complete. As long as the sequence reference database will not be taxonomically covered, the best taxa candidates can not all be interpreted as strictly confident records of the diet. We draw the attention of the reader that this approach, at this stage, only provides a list of hypothetical but likely plant taxa that can be eaten by tapirs.

Nevertheless, our study case in the lowland tapir showed that the resulting information on the diet is well consistent with that from classical methods. It confirmed the consumption of many taxa, extended the range of use of some others and also allowed to discover new likely ones. Our study also showed that further development of the approach and refining of the taxonomical resolution is also possible thanks to the combination of complementary markers.

The genetic reference databases are still under development, including DNA sequences from new organisms in new regions of the world (e.g. [57]). As more taxa are being sequenced a refined appreciation of the composition and richness of the diets should be possible.

In the meantime, we feel that the metabarcoding of faecal DNA can already complement the classical approaches of the diet of tropical herbivores in a non-invasive way. Telemetry studies and analysis of the diet by stable isotopes can help focusing on the areas and habitats and large plant categories (e.g. [58], [59]) in which elusive rainforest mammals forage. If the fruit part of the diet can be well analysed up to the genus and species levels through macroscopic identification of the diasporas residuals in the faeces, the barcoding approach has the advantage to point to many likely taxa composing the less inventoried browsed part of the diet of the rainforest mammals. This can be up to 100% of the diet in tapirs during some periods of the year [18]). The barcoding approach can both extend and refine the list of items compared to classical visual histological approaches (see review in Yoccoz [60]). To date, the browse part in tapir diet has been investigated with best taxonomic resolution by the botanical analysis of browsing signs. Browsing signs can be attributed to tapirs thanks to their characteristic foot prints but in lighter animals not letting identifiable tracks around the found browsed plants, the identification of the browser may be more problematic. In certain cases, the plants may also have been browsed to such an extent that they are no more identifiable nor visible. The faecal DNA barcoding can interestingly complement the information on the taxa browsed by the rainforest animals.

Hence it appears promising to identify key resources and associated habitats for endangered species, better characterize the dynamics of the resource partitioning in elusive herbivore communities, and more generally better appreciate the role of the herbivores in the functioning and the diversity of the rainforest ecosystem. However, we stress that similar tests using diet information from other approaches should be conducted in ruminants and fore-gut fermenters in which the digestive efficiency of fibres is different than in the hind-gut fermenter tapirs [13]. This could also be tested in other endangered groups of elusive species of the rainforest, such as primates and birds, in which information about the diet has been gathered through classic approaches [61] to extend it to groups in which information on the diet is still missing.

### Data accessibility

Fasta files and filtered data have been deposited in the Dryad repository: doi: 10.5061/dryad.8h7k1.

## Supporting Information

Table S1 List of plant taxa identified in tapir faeces of the Nouragues Reserve after matching of the P6 loop of trnL (UAA) sequences with the EMBL reference and local databases. "Indet." stands for indeterminate. The figures into brackets indicate the number of different sequences for the unidentified genera. Species names followed by "?" have been inferred *a posteriori* for genera with only one species listed in the Reserve (www.nouragues.cnrs.fr/). All of these genera but *Bagassa*, however, have several species listed in French Guiana and in the region. Presence of the taxa in the region was checked in Funk *et al.* (2007). Only one species of *Machaerium* has been listed in the Nouragues but two different sequences were found in the tapir dung.
(DOCX)

## References

[1] Redford KH (1992) The empty forest. BioScience 42: 412–422.

[2] Corlett RT (2007) The impact of hunting on the mammalian fauna of tropical Asian forests. Biotropica 39: 292–303.

[3] Terborgh J (1988) The big things that run the world-a sequel to E G Wilson. Conservation Biology 2: 402–403.

[4] Dirzo R, Miranda A (1990) Contemporary neotropical defaunation and forest structure, function, and diversity - a sequel to John Terborgh. Conserv Biol 4: 444–447.

[5] Wright SJ, Zeballos H, Dominguez I, Gallard MM, Moreno M, et al (2000) Poachers alter mammal abundance, seed dispersal, and seed predation in a Neotropical forest. Conserv Biol. 14: 227–239.

[6] Wright SJ, Duber HC (2001) Poachers and forest fragmentation alter seed dispersal, seed survival, and seedling recruitment in the palm *Attalea bufyraceae* with implications for tropical tree diversity. Biotropica 33: 583–595.

[7] Stoner KE, Riba-Hernández P, Vulinec K, Lambert JE (2007) The role of mammals in creating and modifying seedshadows in tropical forests and some possible consequences of their elimination. Biotropica 39: 316–327.

[8] Taber A, Chalukian SC, Altrichter M, Minkowski K, Lizarraga L, et al.. (2008) El destino de los arquitectos de los bosques neotropicales: evaluación de la distribución y el estado de conservación de los pecaríes labiados y los tapires de tierras bajas. New York, NY:Pigs, Peccaries and Hippos Specialist Group (IUCN/SSC), Tapir Specialist Group (IUCN/SSC), Wildlife Conservation Society, Wildlife Trust. 181 p.

[9] Terborgh J, Nuñez-Iturri N, Pitman NCA, Cornejo Valverde, FH, Alvarez P, et al (2008) Tree recruitment in an empty forest. Ecology 89: 1757–1768. 18589539 10.1890/07-0479.1

[10] Fragoso JMV, Huffman JM (2000) Seed-dispersal and seedling recruitment patterns by the last Neotropical megafaunal element in Amazonia, the tapir. J Trop Ecol 16: 369–385.

[11] Moran C, Catterall CP, Kanowski J (2009) Reduced dispersal of native plant species as a consequence of the reduced abundance of frugivore species in fragmented rainforest. Biol Conserv 142(3): 541–552.

[12] Salas LA, Fuller TK (1996) Diet of the lowland tapir (*Tapirus terrestris* L) in the Tabaro River Valley, southern Venezuela. Can J Zool 74: 1444–1451.

[13] Bodmer RE (1991) Influence of digestive morphology on resource partitioning in Amazonian ungulates. Oecologia 85(3): 361–365. 28312040 10.1007/BF00320611

[14] Henry O, Feer F, Sabatier D (2000) Diet of the lowland tapir (Tapirus terrestris) in French Guiana. Biotropica 3: 364–368.

[15] Gayot M, Henry O, Dubost G, Sabatier D (2004) Diet of the two forest cervids of the genus Mazama in French Guiana. J Trop Ecol 20: 31–43.

[16] Steinheim G, Wegge P, Fjellstad JI, Jnawali SR, Weladji RB (2005) Dry season diets and habitat use of sympatric Asian elephants (*Elephas maximus*) and greater one-horned rhinoceros (*Rhinocerus unicornis*) in Nepal. J Zool 265: 377–385.

[17] Tobler MW, Janovec JP, Cornejo F (2010) Frugivory and seed dispersal by the lowland tapir Tapirus terrestris in the Peruvian Amazon. Biotropica 42: 215–222.

[18] Hibert F, Sabatier D, Andrivot J, Scotti-Saintagne C, Gonzalez S, et al (2011) Botany, genetics and ethnobotany: a crossed investigation on the elusive tapir's diet in French Guiana. PLoS ONE 6(10): e25850. 21991372 10.1371/journal.pone.0025850 PMC3185057

[19] Steinmetz R, Chutipong W, Seuaturien N, Chirngsaard E (2008) Community Structure of Large Mammals in Tropical Montane and Lowland Forest in the Tenasserim-Dawna Mountains, Thailand. Biotropica 40: 344–353.

[20] Valentini A, Pompanon F, Taberlet P (2009a) DNA barcoding for ecologists. Trends Ecol Evol 24: 110–117. 19100655 10.1016/j.tree.2008.09.011

[21] Rayé G, Miquel C, Coissac E, Redjadj C, Loison A, et al (2011) New insights on diet variability revealed by DNA barcoding and high-throughput pyrosequencing: chamois diet in autumn as a case study. Ecological Research 26(2): 265–276.

[22] Shehzad W, Riaz T, Nawaz MA, Miquel C, Poillot C, et al (2012a) Carnivore diet analysis based on next-generation sequencing: application to the leopard cat (Prionailurus bengalensis) in Pakistan. Mol Ecol 21: 1951–1965. 22250784 10.1111/j.1365-294X.2011.05424.x

[23] Floyd R, Abebe E, Papert A, Blaxter M (2002) Molecular barcodes for soil nematode identification. Mol Ecol 11: 839–850. 11972769 10.1046/j.1365-294x.2002.01485.x

[24] Valentini A, Miquel C, Nawaz MA, Bellemain E, Coissac E, et al (2009b) New perspectives in diet analysis based on DNA barcoding and parallel pyrosequencing: the trnL approach. Mol Ecol Resources 9: 51–60. 10.1111/j.1755-0998.2008.02352.x 21564566

[25] Deagle BE, Kirkwood R, Jarman SN (2009) Analysis of Australian fur seal diet by pyrosequencing prey DNA in faeces. Mol Ecol 18: 2022–2038. 19317847 10.1111/j.1365-294X.2009.04158.x

[26] Deagle BE, Chiaradia A, McInnes J, Jarman SN (2010) Pyrosequencing faecal DNA to determine diet of little penguins: is what goes in what comes out? Conservation Genetics 11: 2039–2048.

[27] Shehzad W, McCarthy TM, Pompanon F, Purevjav L, Coissac E, et al (2012b) Prey Preference of Snow Leopard (Panthera uncia) in South Gobi, Mongolia. PLoS ONE 7(2): e32104. 22393381 10.1371/journal.pone.0032104 PMC3290533

[28] Pegard A, Miquel C, Valentini A, Coissac E, Bouvier F, et al (2009) Universal DNA based methods for assessing the diet of grazing livestock and wildlife from faeces. J Agric Food Chem 57: 5700–5706. 19566081 10.1021/jf803680c

[29] Soininen EM, Valentini A, Coissac E, Miquel C, Gielly L, et al.. (2009) Analysing diet of small herbivores: the efficiency of DNA barcoding coupled with high-throughput pyrosequencing for deciphering the composition of complex plant mixtures. Front Zool 6(16). 10.1186/1742-9994-6-16 PMC2736939 19695081

[30] Kowalczyk R, Taberlet P, Coissac E, Valentini A, Miquel C, et al (2011) Influence of management practices on large herbivore diet — Case of European bison in Białowieża Primeval Forest (Poland). For Ecol Manage 261(4): 821–828.

[31] Baamrane MAA, Shehzad W, Ouhammou A, Abbad A, Naimi M, et al (2012) Assessment of the food habits of the Moroccan dorcas gazelle in M'Sabih Talaa, West central Morocco, using the trnL approach. PLoS One 7: e35643. 22558187 10.1371/journal.pone.0035643 PMC3338736

[32] Navarro SP, Jurado-Rivera JA, Gómez-Zurita J, Lyal CHC, Vogler AP (2010) DNA profiling of host-herbivore interactions in tropical forests. Ecol Entomol 35: 18–32.

[33] Bradley BJ, Stiller M, Doran-Sheehy DM, Harris T, Chapman CA, et al (2007) Plant DNA sequences from feces: potential means for assessing diets of wild primates. Am J Primatol 69: 699–705. 17216626 10.1002/ajp.20384

[34] Taberlet P, Coissac E, Pompanon F, Gielly L, Miquel C, et al (2007) Power and limitations of the chloroplast trnL(UAA) intron for plant DNA barcoding. Nucleic Acids Res 35: e14. 17169982 10.1093/nar/gkl938 PMC1807943

[35] Kress WJ, Wurdack KJ, Zimmer EA, Weigt LA, Janzen DH (2005) Use of DNA barcodes to identify flowering plants. Proc Natl Acad Sci USA 102: 8369–8374. 15928076 10.1073/pnas.0503123102 PMC1142120

[36] Taberlet P, Coissac E, Hajibabaei M, Rieseberg LH (2012) Environmental DNA. Mol Ecol 21: 1789–1793. 22486819 10.1111/j.1365-294X.2012.05542.x

[37] Yoccoz NG, Bråthen KA, Gielly L, Haile J, Edwards ME, et al (2012) DNA from soil mirrors plant taxonomic and growth form diversity. Mol Ecol 21: doi: 10.1111/j.1365-1294X.2012.05545.x. 10.1111/j.1365-294X.2012.05545.x 22507540

[38] Gonzalez MA, Baraloto C, Engel J, Mori SA, Pétronelli P, et al (2009) Identification of Amazonian trees with DNA barcodes. PLoS ONE 4: 7483. 10.1371/journal.pone.0007483 PMC2759516 19834612

[39] Bongers F, Charles-Dominique P, Forget PM, Théry M (2001) Nouragues: Dynamics and plant animal interactions in a Neotropical rainforest. Dordrecht, Netherlands: Kluwer Academic Publishers. 456 p.

[40] Mori SA, Cremers G, Gracie C, de Granville JJ, Heald SV, et al.. (2002) Guide to the vascular plants of central French Guiana. Part 2. Dicotyledons. New York, NY: New York Botanical Garden Press.

[41] Funk V, Hollowell T, Berry P, Kelloff C, Alexander SN (2007) Checklist of the plants of the Guiana Shield (Venezuela: Amazonas, Bolivar, Delta Amacuro; Guyana, Surinam, French Guiana) Smithsonian Institution, Contributions from the United States National Herbarium. 55: 1–584.

[42] Sabatier D (1985) Saisonnalité et déterminisme du pic de fructification en forêt guyanaise. Revue d'Ecologie (la Terre et la Vie) 40: 289–320.

[43] Norden N, Chave J, Belbenoit P, Caubère A, Châtelet P, et al (2007) Mast fruiting is a frequent strategy in woody species of eastern south America. PLoS ONE 2: e1079. 17957261 10.1371/journal.pone.0001079 PMC2031917

[44] Needleman SB, Wunsch CD (1970) A general method applicable to the search for similarities in the amino acid sequence of two proteins. J Mol Biol 48: 443–453. 5420325 10.1016/0022-2836(70)90057-4

[45] Ficetola GF, Coissac E, Zundel S, Riaz T, Shehzad W, et al (2010) An in silico approach for the evaluation of DNA barcodes. BMC Genomics 11: 434. 20637073 10.1186/1471-2164-11-434 PMC3091633

[46] Tuomisto H (2010) A diversity of beta diversities: straightening up a concept gone away. Part 1. Defining beta diversity as a function of alpha and gamma diversity. Ecography 33: 2–22.

[47] Colwell RK, Mao CX, Chang J (2004) Interpolating, extrapolating, and comparing incidence-based species accumulation curves. Ecology 85: 2717–2727.

[48] Colwell RK (2009) EstimateS: Statistical estimation of species richness and shared species from samples. Version 8.2. User's Guide and application published at: http://purl.oclc.org/estimates, accessed on the 6/12/2010.

[49] Walther BA, Moore JL (2005) The concepts of bias, precision and accuracy, and their use in testing the performance of species richness estimators, with a literature review of estimator performance. Ecography 28: 815–829.

[50] Dolédec S, Chessel D (1987) Rythmes saisonniers et composantes stationnelles en milieu aquatique I - Description d'un plan d'observations complet par projection de variables. Acta Oecologica, Oecologia Generalis 8(3): 403–426.

[51] Tobler MW (2008) The ecology of the lowland tapir in Madre de Dios, Peru: using new technologies to study large rainforest mammals. College Station, Texas: PhD Dissertation. Texas A&M University. 132 p.

[52] Poncy O, Sabatier D, Prévost M-F, Hardy I (2001) Chapter 4: The lowland high rainforest: structure and tree species diversity. In : Bongers F, Charles-Dominique P, Forget P-M, Théry M, editors. Nouragues: dynamics and plant animal interactions in a neotropical rainforest’. Kluwer Academic Publishers, Dordreht, Boston. pp. 31-46.

[53] Dray S, Dufour AB (2007) The ade4 package: implementing the duality diagram for ecologists. Journal of Statistical Software 22(4): 1–20.

[54] Newmaster SG, Fazekas AJ, Ragupathy S (2006) DNA barcoding in land plants: evaluation of rbcL in a multigene tiered approach. Canadian Journal of Botany 84: 335–341.

[55] Zhang Q, Liu Y (2003) Sodmergen (2003) Examination of the cytoplasmatic DNA in male reproductive cells to determine the potential for cytoplasmatic inheritance in 295 angiosperm species. Plant Cell Physiol 44: 941–951. 14519776 10.1093/pcp/pcg121

[56] Alcover JA, Perez-Obiol R, Yll EI, Bover P (1999) The diet of *Myotragus balearicus* Bate 1909 (Artiodactyla: Caprinae), an extinct bovid from the Balearic Islands: evidence from coprolites. Biological Journal of the Linnean Society 66(1): 57–74.

[57] Lim J, Kim S-Y, Kim S, Eo HS, Kim CB, et al.. (2009) BioBarcode: a general DNA barcoding database and server platform for Asian biodiversity resources. BMC Genomics (Suppl 3):S8. 10.1186/1471-2164-10-S3-S8 PMC2788395 19958506

[58] Cerling TE, Hart JA, Hart TB (2004) Stable isotope ecology in the Ituri Forest. Oecologia 138(1): 5–12. 14530961 10.1007/s00442-003-1375-4

[59] DeSantis LG (2011) Stable isotope ecology of extant tapirs from the Americas. Biotropica 43: 746–754.

[60] Yoccoz NG (2012) The future of environmental DNA in ecology. Mol Ecol 21: 2031–2038. 22486823 10.1111/j.1365-294X.2012.05505.x

[61] Bradford MG, Dennis AJ, Westcott DA (2008) Diet and Dietary Preferences of the Southern Cassowary (*Casuarius casuarius*) in North Queensland, Australia. Biotropica 40: 338–343.

