# Supplementary material for: Unveiling the Diet of Elusive Rainforest Herbivores in Next Generation Sequencing Era? The Tapir as a Case Study
Source: PLoS One. 2013 Apr 1;8(4):e60799. doi: 10.1371/journal.pone.0060799 (PMC3613382; doi:10.1371/journal.pone.0060799)
Supplement: Table S1 — List of plant taxa identified in tapir faeces of the Nouragues Reserve after matching of the P6 loop of trnL (UAA) sequences with the EMBL reference and local databases. "Indet." stands for indeterminate. The figures into brackets indicate the number of different sequences for the unidentified genera. Species names followed by "?" have been inferred a posteriori for genera with only one species listed in the Reserve (www.nouragues.cnrs.fr/). All of these genera but Bagassa, however, have several species listed in French Guiana and in the region. Presence of the taxa in the region was checked in Funk et al. (2007). Only one species of Machaerium has been listed in the Nouragues but two different sequences were found in the tapir dung. (DOCX) [file pone.0060799.s001.docx]

**Table S1**:

| Family | Genus (number of sequences) | Species |
| --- | --- | --- |
| Acanthaceae | indet. (1) |  |
| Anacardiaceae | Spondias | S. mombin Linnaeus |
|  | Tapirira |  |
| Annonaceae | indet. (1) |  |
| Apocynaceae | Geissospermum |  |
|  | indet. (5) |  |
| Araceae | indet. (2) |  |
| Arecaceae | indet. (2) |  |
| Bignoniaceae | indet. (4) |  |
| Bromeliaceae | Aechmea |  |
| Burseraceae | indet. (2) |  |
| Caricaceae | indet. (1) | Jacaratia spinosa (J.B. Aublet) A.P. De Candolle? |
| Celastraceae | indet. (3) |  |
| Chrysobalanaceae | indet. (1) |  |
| Combretaceae | Terminalia |  |
| Convolvulaceae | indet. (1) |  |
| Cucurbitaceae | indet. (3) |  |
| Cyclanthaceae | indet. (3) |  |
| Euphorbiaceae | Hieronyma | Hieronyma alchorneoides |
| Fabaceae | Dalbergia | D. monetaria Linnaeus f. ? |
|  | Eperua |  |
|  | Inga |  |
|  | Machaerium (2) | M. inundatum (Martius ex Bentham) Ducke ? + M. sp. ? |
|  | Senna |  |
|  | Vouacapoua | V. americana J.B. Aublet ? |
|  | indet (9) |  |
| Gesneriaceae | indet (1) |  |
| Humiriaceae | Vantanea | V. parviflora Lamarck ? |
| Lecythidaceae | indet. (1) |  |
| Loganiaceae | Strychnos |  |
| Malpighiaceae | indet. (1) |  |
| Marattiaceae | Danaea |  |
| Marcgraviaceae | Norantea |  |
| Melastomataceae | Adelobotrys |  |
|  | Miconia |  |
|  | Mouriri |  |
|  | indet (1) |  |
| Meliaceae | Carapa |  |
|  | indet (1) |  |
| Moraceae | Bagassa | B. guianensis J.B. Aublet ? |
|  | Brosimum |  |
|  | Ficus |  |
|  | indet. (3) |  |
| Myristicaceae | indet. (1) |  |
| Ochnaceae | Lacunaria |  |
| Onagraceae | Ludwigia | L. hyssopifolia (G. Don) Exell |
| Piperaceae | Piper |  |
| Polygalaceae | indet. (3) |  |
| Polygonaceae | indet. (1) |  |
| Rubiaceae | Argostemma |  |
|  | Carapichea | Carapichea guianensis J.B. Aublet? |
|  | Chimarrhis | C. turbinata De Candolle ? |
|  | Coussarea |  |
|  | Faramea |  |
|  | Psychotria |  |
|  | indet (6) |  |
| Sapindaceae | indet. (3) |  |
| Sapotaceae | Ecclinusa |  |
|  | Pradosia |  |
|  | indet. (1) |  |
| Solanaceae | Solanum |  |
|  | indet. (2) |  |
| Urticaceae | Pourouma |  |
|  | indet (1) |  |
| Violaceae | Amphirrhox | A. longifolia (Saint-Hilaire) K.P. Sprengel ? |
| Vochysiaceae | Erisma | E. uncinatum Warming ? |
|  | Vochysia |  |
|  | indet. (1) |  |
|  |  |  |
